# Crystal structure of bis­{*S*-octyl-3-[(thio­phen-2-yl)methyl­idene]di­thio­carbazato-κ^2^ *N* ^3^,*S*}nickel(II)

**DOI:** 10.1107/S2056989023005935

**Published:** 2023-07-11

**Authors:** Sultana Shakila Khan, Md. Belayet Hossain Howlader, Md. Chanmiya Sheikh, Ryuta Miyatake, Ennio Zangrando

**Affiliations:** a Department of Chemistry, Rajshahi University, Rajshahi-6205, Bangladesh; b Department of Applied Science, Faculty of Science, Okayama University of Science, Japan; c Center for Environmental Conservation and Research Safety, University of Toyama, 3190 Gofuku, Toyama, 930-8555, Japan; d Department of Chemical and Pharmaceutical Sciences, University of Trieste, Italy; University of Kentucky, USA

**Keywords:** crystal structure, nickel(II) complex, di­thio­carbazato ligand

## Abstract

In the title complex, [Ni(C_14_H_21_N_2_S_3_)_2_], the nickel(II) atom is located on a crystallographic inversion center and exhibits a square-planar coordination environment, being coordinated by two negatively charged N,S*-*chelating ligands in a *trans* configuration. In the crystal, the non-H atoms of the complex are practically coplanar (r.m.s. deviation of fitted atoms = 0.135 Å), and the angle between the thienyl and the chelating rings is 6.7 (1)°. The mol­ecules stack at a distance of 3.623 (2) Å along the *b*-axis direction.

## Abstract 2

The mononuclear nickel(II) complex is bis-chelated by di­thio­carbazato ligands bearing a thienyl ring and an *n*-octyl alkyl chain.

## 1. Chemical context

Thio­semicarbazones, semicarbazones, hydrazide/hydrazones and di­thio­carbazate ligands have been widely employed for the preparation of metal complexes. Over the last few decades, di­thio­carbazate Schiff bases and their metal complexes have gained considerable inter­est because of their promising bioactivities against diverse cancer cell lines (Yusof *et al.*, 2015 ▸; Ramilo-Gomes *et al.*, 2021 ▸; Low *et al.*, 2016 ▸), as well as anti­microbial activity (Zangrando *et al.*, 2017 ▸). Clearly, the biological properties of these compounds can be modulated by using different organic substituents, leading to concomitant structural modifications (How *et al.*, 2008 ▸; Yusof *et al.*, 2022 ▸). A study of structure–activity relationships was described by Beshir *et al.* (2008 ▸). 
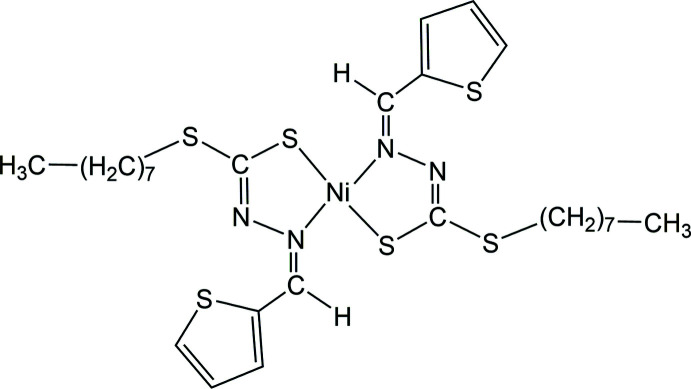


**Figure 1:**
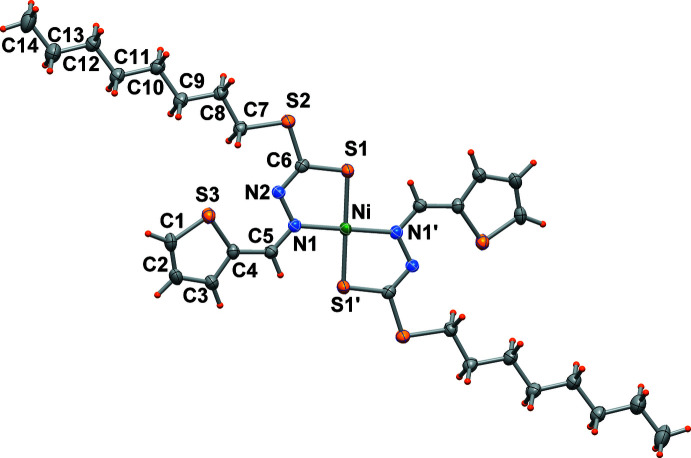
An ellipsoid plot (50% probability) of the title compound.

Therefore, considering the diverse significance of di­thio­carbazate bases and their role in a variety of biological applications, herein we report a novel Ni^II^ complex with a di­thio­carbazate Schiff base ligand bearing an octyl alkyl chain and a thienyl ring (Fig. 1 ▸).

## 2. Structural commentary

The nickel(II) atom is located on a crystallographic center of symmetry and exhibits a square-planar coordination sphere, being coordinated by two negatively charged N,S-chelating ligands in a *trans* configuration. The Ni—N1 and Ni—S1 bond distances are 1.9168 (19) and 2.1735 (7) Å, respectively with a chelating N1—Ni—S1 bond angle of 85.88 (6)°. These values agree with those reported in previous papers (Begum *et al.*, 2016 ▸; Islam *et al.*, 2014 ▸; Howlader *et al.*, 2015 ▸) for related compounds. It is worth mentioning that nickel(II) and copper(II) complexes with di­thio­carbazate ligands have been reported to crystallize in both *cis* and *trans* configurations, although the latter is slightly more frequent (Begum *et al.*, 2020 ▸).

All of the non-H atoms of the complex are almost coplanar, with S1 and C1 [−0.28 Å] and C13, C14 [+0.24, +0.31 Å], respectively deviating the most from its mean plane (r.m.s. deviation of fitted atoms = 0.135 Å). The thienyl ring forms a small dihedral angle of 6.7 (1)° with respect to the chelating five-membered ring. The long alkyl chain is in a staggered conformation with torsion angles along the chain that range between 176.7 (2) and 179.8 (2)°.

The mol­ecule is stabilized by an intra­molecular unconventional hydrogen bond between C5—H5 with S1′ [at 1 − *x*, 1 − *y*, 1 − *z*] of the symmetry-related ligand [C5⋯S1′ distance of 3.067 (3) Å, C5—H5⋯S1′ angle of 125°].

## 3. Supra­molecular features

**Figure 2:**
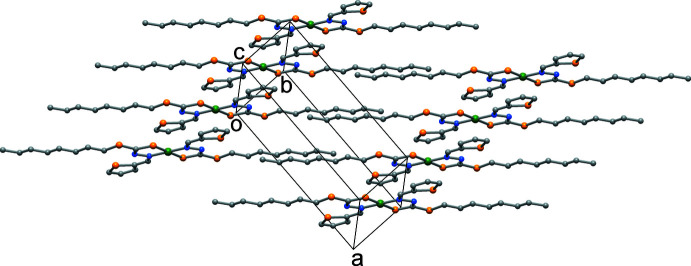
A partial packing view showing complexes stacked in the *b*-axis direction.

**Figure 3:**
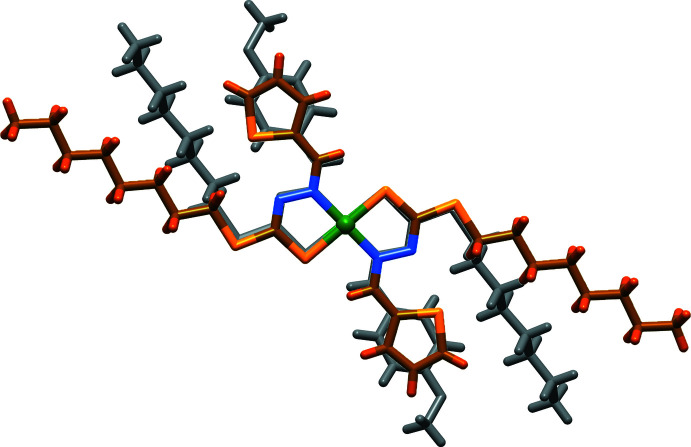
Superposition of this structure with the 4-meth­oxy­benzyl derivative WEGKEB (Begum *et al.*, 2018 ▸; only one disorder component shown), where it is worth noting the different orientation of the octyl moiety, likely induced by crystal-packing requirements.

The mol­ecules stack with an inter­planar distance of 3.623 (2) Å, and the crystal packing shows that all hydro­phobic *n*-octyl chains segregate together, so as to share the same regions of space (Fig. 2 ▸), as already observed in similar complexes (Begum *et al.*, 2016 ▸). Fig. 3 ▸ overlays this structure of the complex superimposed onto that of a 4-meth­oxy­benzyl derivative (WEGKEB: Begum *et al.*, 2018 ▸), where it is worth noting the different orientation of octyl chains in the two cases. This is due to the different torsion angle C6—S2—C7—C8 of −177.36 (18)° in this structure *vs* 86.8 (6)° and −160.0 (9)° (for the two disorder components of the equivalent torsion angle in WEGKEB), likely induced by crystal-packing requirements. Details of hydrogen-bonding inter­actions are given in Table 1 ▸.

## 4. Database survey

For comparison, Ni^II^ complexes with comparable ligands bearing long alkyl chains have been reported from these laboratories (Begum *et al.*, 2016 ▸, 2017 ▸, 2018 ▸, 2020 ▸, 2023 ▸; CSD refcodes = JUYCAJ, WEGKEB, BIQTIH, TILVUJ and PICMOH, respectively).

## 5. Synthesis and crystallization

A solution of Ni(CH_3_COO)_2_·4H_2_O (0.12 g, 0.5 mmol in 10 mL methanol) was added to a solution of *S*-octyl-β-*N*-(2-thien­yl)methyl­enedi­thio­carbazate (0.314 g, 1.0 mmol in 30 mL of methanol). The resulting mixture was stirred at room temperature for 4 h. The dark-orange precipitate that formed was filtered off, washed with methanol and dried *in vacuo* over anhydrous CaCl_2_. Orange needle-shaped single crystals, suitable for X-ray diffraction, were obtained by slow evaporation of the compound from a mixture of chloro­form and aceto­nitrile (4:1, *v*/*v*) after 14 days. Yield: 66%; m. p. (377-378) K.

FT–IR (KBr, cm^−1^): 2920 ν(C—H, alk­yl), 1639, 1572 ν(C=N—N=C).

^1^H NMR (400 MHz, CDCl_3_, ppm) δ: 7.999 (*s*, 2×1H, CH=N, C-5), 7.715 (*d*, 2×1H, C-1, *J* = 5.2 Hz), 7.468 (*d*, 2×1H, C-3, *J* = 5.2 Hz), 7.103 (*t*, 2×1H, C-2), 3.269 (*t*, 2×2H, –SCH_2_, C-7), 1.764 (*p*, 2×2H, C-8), 1.460 (*p*, 2×2H, C-9), 1.318–1.270 (*m*, 2×8H, C-10, 11, 12, 13), 0.878 (*t*, 2×3H, C-14).

UV–Vis spectrum [CHCl_3_, λ_max_ nm]: 475, 400, 276.

HRMS (FAB) Calculated for C_28_H_42_N_4_NiS_6_ [*M*+H]^+^: 685.11599, found [*M*+H]^+^: 685.11549.

## 6. Refinement

Crystal data, data collection and structure refinement are summarized in Table 2 ▸. Hydrogen atoms were placed at calculated positions (C–H = 0.95–0.99 Å) and refined as riding with *U*_iso_(H) = 1.2–1.5*U*_eq_(C).

## Supplementary Material

Crystal structure: contains datablock(s) global, I. DOI: 10.1107/S2056989023005935/pk2690sup1.cif

Structure factors: contains datablock(s) I. DOI: 10.1107/S2056989023005935/pk2690Isup2.hkl

CCDC reference: 2254902

Additional supporting information:  crystallographic information; 3D view; checkCIF report

## Figures and Tables

**Table 1:** Hydrogen-bond geometry (Å, °)

*D*—H⋯*A*	*D*—H	H⋯*A*	*D*⋯*A*	*D*—H⋯*A*
C2—H2⋯S1^i^	0.95	3.00	3.684 (3)	131
C2—H2⋯S2^ii^	0.95	2.93	3.752 (3)	146
C5—H5⋯S1^iii^	0.95	2.42	3.067 (3)	125
C7—H7*A*⋯S3	0.99	2.93	3.406 (3)	110

Symmetry codes: (i) 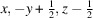 ; (ii) 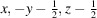 ; (iii) 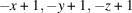 .

**Table 2:** Experimental details

Crystal data	
Chemical formula	[Ni(C_14_H_21_N_2_S_3_)_2_]
*M*_r_	685.72
Crystal system, space group	Monoclinic, *P*2_1_/*c*
Temperature (K)	173
*a*, *b*, *c* (Å)	15.5444 (6), 5.5388 (3), 20.1592 (8)
β (°)	103.675 (7)
*V* (Å^3^)	1686.44 (13)
*Z*	2
Radiation type	Mo *K*α
μ (mm^−1^)	0.97
Crystal size (mm)	0.08 × 0.02 × 0.01

Data collection	
Diffractometer	Rigaku R-AXIS RAPID
Absorption correction	Multi-scan (*ABSCOR*; Rigaku, 1995 ▸)
*T*_min_, *T*_max_	0.815, 0.990
No. of measured, independent and observed [*I* > 2σ(*I*)] reflections	15791, 3850, 2621
*R*_int_	0.077
(sin θ/λ)_max_ (Å^−1^)	0.649

Refinement	
*R*[*F* ^2^ > 2σ(*F* ^2^)], *wR*(*F* ^2^), *S*	0.045, 0.079, 1.00
No. of reflections	3850
No. of parameters	179
H-atom treatment	H-atom parameters constrained
Δρ_max_, Δρ_min_ (e Å^−3^)	0.44, −0.27

Computer programs: *RAPID-AUTO* (Rigaku, 2018 ▸), *SHELXT* (Sheldrick, 2015*a* ▸), *SHELXL2019/2* (Sheldrick, 2015*b* ▸), *DIAMOND* (Brandenburg, 1999 ▸) and *WinGX* (Farrugia, 2012 ▸).

## supplementary crystallographic information

### Crystal data

**Table:**

[Ni(C_14_H_21_N_2_S_3_)_2_]	*F*(000) = 724
*M**_r_* = 685.72	*D*_x_ = 1.350 Mg m^−^^3^
Monoclinic, *P*2_1_/*c*	Mo *K*α radiation, λ = 0.71075 Å
*a* = 15.5444 (6) Å	Cell parameters from 10434 reflections
*b* = 5.5388 (3) Å	θ = 2.1–27.5°
*c* = 20.1592 (8) Å	µ = 0.97 mm^−^^1^
β = 103.675 (7)°	*T* = 173 K
*V* = 1686.44 (13) Å^3^	Needle, orange
*Z* = 2	0.08 × 0.02 × 0.01 mm

### Data collection

**Table:**

Rigaku R-AXIS RAPID diffractometer	2621 reflections with *I* > 2σ(*I*)
Detector resolution: 10.000 pixels mm^-1^	*R*_int_ = 0.077
ω scans	θ_max_ = 27.5°, θ_min_ = 2.7°
Absorption correction: multi-scan (ABSCOR; Rigaku, 1995)	*h* = −19→20
*T*_min_ = 0.815, *T*_max_ = 0.990	*k* = −7→7
15791 measured reflections	*l* = −26→25
3850 independent reflections	

### Refinement

**Table:**

Refinement on *F*^2^	0 restraints
Least-squares matrix: full	Hydrogen site location: inferred from neighbouring sites
*R*[*F*^2^ > 2σ(*F*^2^)] = 0.045	H-atom parameters constrained
*wR*(*F*^2^) = 0.079	*w* = 1/[σ^2^(*F*_o_^2^) + (0.0329*P*)^2^ + 0.0128*P*] where *P* = (*F*_o_^2^ + 2*F*_c_^2^)/3
*S* = 1.00	(Δ/σ)_max_ = 0.001
3850 reflections	Δρ_max_ = 0.44 e Å^−^^3^
179 parameters	Δρ_min_ = −0.27 e Å^−^^3^

### Special details

**Table:**

Geometry. All esds (except the esd in the dihedral angle between two l.s. planes) are estimated using the full covariance matrix. The cell esds are taken into account individually in the estimation of esds in distances, angles and torsion angles; correlations between esds in cell parameters are only used when they are defined by crystal symmetry. An approximate (isotropic) treatment of cell esds is used for estimating esds involving l.s. planes.

### Fractional atomic coordinates and isotropic or equivalent isotropic displacement parameters (Å^2^)

**Table:**

	*x*	*y*	*z*	*U*_iso_*/*U*_eq_	
Ni1	0.500000	0.500000	0.500000	0.02461 (12)	
S1	0.43249 (4)	0.34173 (13)	0.57239 (3)	0.03295 (17)	
S2	0.30020 (4)	−0.04469 (12)	0.55073 (3)	0.03283 (17)	
S3	0.33321 (4)	−0.17114 (13)	0.33566 (3)	0.03364 (17)	
N1	0.43677 (12)	0.2681 (4)	0.43617 (10)	0.0263 (5)	
N2	0.37776 (13)	0.1034 (4)	0.45452 (10)	0.0274 (5)	
C1	0.32299 (17)	−0.2689 (5)	0.25377 (13)	0.0364 (7)	
H1	0.290649	−0.408991	0.235505	0.044*	
C2	0.36579 (17)	−0.1259 (5)	0.21773 (13)	0.0384 (7)	
H2	0.366185	−0.152919	0.171263	0.046*	
C3	0.40956 (16)	0.0668 (5)	0.25668 (12)	0.0331 (6)	
H3	0.443110	0.184342	0.239444	0.040*	
C4	0.39872 (15)	0.0677 (4)	0.32296 (12)	0.0273 (6)	
C5	0.44139 (15)	0.2400 (5)	0.37290 (12)	0.0289 (6)	
H5	0.478965	0.351305	0.357540	0.035*	
C6	0.37280 (14)	0.1323 (4)	0.51739 (12)	0.0255 (5)	
C7	0.25455 (16)	−0.2377 (5)	0.47858 (12)	0.0317 (6)	
H7A	0.228117	−0.136545	0.438453	0.038*	
H7B	0.302730	−0.334361	0.467263	0.038*	
C8	0.18415 (17)	−0.4070 (5)	0.49347 (13)	0.0338 (6)	
H8A	0.210547	−0.512821	0.532553	0.041*	
H8B	0.136169	−0.311795	0.505600	0.041*	
C9	0.14665 (17)	−0.5596 (5)	0.43057 (13)	0.0364 (7)	
H9A	0.123844	−0.450452	0.391478	0.044*	
H9B	0.195365	−0.655482	0.419806	0.044*	
C10	0.07291 (17)	−0.7307 (5)	0.43757 (13)	0.0368 (6)	
H10A	0.025185	−0.637168	0.450618	0.044*	
H10B	0.096283	−0.847621	0.474588	0.044*	
C11	0.03444 (17)	−0.8679 (5)	0.37195 (13)	0.0393 (7)	
H11A	0.011898	−0.749798	0.335090	0.047*	
H11B	0.082584	−0.960448	0.359172	0.047*	
C12	−0.03979 (17)	−1.0408 (5)	0.37605 (14)	0.0403 (7)	
H12A	−0.016871	−1.163148	0.411632	0.048*	
H12B	−0.087282	−0.949827	0.390228	0.048*	
C13	−0.0790 (2)	−1.1686 (7)	0.30929 (15)	0.0569 (9)	
H13A	−0.102651	−1.046174	0.273913	0.068*	
H13B	−0.031228	−1.257274	0.294768	0.068*	
C14	−0.1521 (2)	−1.3442 (7)	0.31334 (18)	0.0693 (11)	
H14A	−0.128788	−1.469718	0.347054	0.083*	
H14B	−0.174872	−1.418856	0.268549	0.083*	
H14C	−0.200158	−1.257687	0.327044	0.083*	

### Atomic displacement parameters (Å^2^)

**Table:**

	*U*^11^	*U*^22^	*U*^33^	*U*^12^	*U*^13^	*U*^23^
Ni1	0.0294 (2)	0.0213 (3)	0.0228 (2)	−0.0016 (2)	0.00554 (18)	−0.00261 (19)
S1	0.0420 (4)	0.0320 (4)	0.0256 (3)	−0.0103 (3)	0.0095 (3)	−0.0052 (3)
S2	0.0415 (4)	0.0294 (4)	0.0287 (3)	−0.0086 (3)	0.0105 (3)	0.0004 (3)
S3	0.0419 (4)	0.0278 (4)	0.0300 (3)	−0.0062 (3)	0.0062 (3)	−0.0026 (3)
N1	0.0299 (11)	0.0220 (12)	0.0270 (11)	−0.0014 (9)	0.0067 (9)	−0.0013 (9)
N2	0.0345 (11)	0.0225 (12)	0.0265 (11)	−0.0050 (9)	0.0098 (9)	−0.0008 (9)
C1	0.0406 (15)	0.0330 (17)	0.0324 (14)	−0.0041 (13)	0.0021 (12)	−0.0101 (12)
C2	0.0391 (15)	0.0460 (19)	0.0297 (13)	−0.0038 (13)	0.0076 (13)	−0.0126 (13)
C3	0.0365 (14)	0.0368 (17)	0.0265 (12)	−0.0044 (12)	0.0087 (12)	−0.0051 (11)
C4	0.0298 (13)	0.0232 (14)	0.0278 (12)	−0.0008 (10)	0.0046 (11)	−0.0025 (10)
C5	0.0327 (13)	0.0260 (15)	0.0298 (13)	−0.0038 (11)	0.0109 (11)	−0.0015 (11)
C6	0.0270 (12)	0.0202 (14)	0.0285 (13)	0.0008 (10)	0.0049 (11)	0.0037 (10)
C7	0.0377 (14)	0.0261 (15)	0.0306 (13)	−0.0045 (11)	0.0068 (12)	−0.0009 (11)
C8	0.0373 (14)	0.0291 (15)	0.0347 (14)	−0.0064 (11)	0.0077 (12)	0.0019 (11)
C9	0.0379 (14)	0.0314 (17)	0.0398 (15)	−0.0074 (12)	0.0091 (13)	−0.0027 (12)
C10	0.0397 (14)	0.0300 (16)	0.0421 (15)	−0.0072 (12)	0.0122 (13)	−0.0020 (12)
C11	0.0396 (15)	0.0370 (18)	0.0405 (15)	−0.0097 (13)	0.0078 (13)	−0.0037 (13)
C12	0.0419 (15)	0.0355 (18)	0.0430 (15)	−0.0089 (13)	0.0090 (13)	−0.0027 (13)
C13	0.0565 (19)	0.060 (2)	0.0522 (19)	−0.0206 (17)	0.0084 (16)	−0.0114 (17)
C14	0.063 (2)	0.062 (3)	0.072 (2)	−0.0261 (19)	−0.0053 (19)	−0.009 (2)

### Geometric parameters (Å, º)

**Table:**

Ni1—N1^i^	1.9168 (19)	C7—H7B	0.9900
Ni1—N1	1.9168 (19)	C8—C9	1.521 (3)
Ni1—S1^i^	2.1735 (7)	C8—H8A	0.9900
Ni1—S1^i^	2.1735 (7)	C8—H8B	0.9900
Ni1—S1	2.1735 (7)	C9—C10	1.519 (3)
S1—C6	1.717 (2)	C9—H9A	0.9900
S2—C6	1.745 (2)	C9—H9B	0.9900
S2—C7	1.809 (2)	C10—C11	1.520 (3)
S3—C1	1.709 (3)	C10—H10A	0.9900
S3—C4	1.725 (3)	C10—H10B	0.9900
N1—C5	1.304 (3)	C11—C12	1.516 (4)
N1—N2	1.404 (3)	C11—H11A	0.9900
N2—C6	1.298 (3)	C11—H11B	0.9900
C1—C2	1.351 (4)	C12—C13	1.515 (4)
C1—H1	0.9500	C12—H12A	0.9900
C2—C3	1.402 (4)	C12—H12B	0.9900
C2—H2	0.9500	C13—C14	1.513 (4)
C3—C4	1.386 (3)	C13—H13A	0.9900
C3—H3	0.9500	C13—H13B	0.9900
C4—C5	1.431 (3)	C14—H14A	0.9800
C5—H5	0.9500	C14—H14B	0.9800
C7—C8	1.524 (3)	C14—H14C	0.9800
C7—H7A	0.9900		
			
N1^i^—Ni1—N1	180.0	C9—C8—H8A	109.8
N1^i^—Ni1—S1^i^	85.88 (6)	C7—C8—H8A	109.8
N1—Ni1—S1^i^	94.12 (6)	C9—C8—H8B	109.8
N1^i^—Ni1—S1^i^	85.88 (6)	C7—C8—H8B	109.8
N1—Ni1—S1^i^	94.12 (6)	H8A—C8—H8B	108.3
S1^i^—Ni1—S1^i^	0.00 (2)	C10—C9—C8	114.7 (2)
N1^i^—Ni1—S1	94.12 (6)	C10—C9—H9A	108.6
N1—Ni1—S1	85.88 (6)	C8—C9—H9A	108.6
S1^i^—Ni1—S1	180.0	C10—C9—H9B	108.6
S1^i^—Ni1—S1	180.0	C8—C9—H9B	108.6
C6—S1—Ni1	96.42 (8)	H9A—C9—H9B	107.6
C6—S2—C7	100.88 (12)	C9—C10—C11	112.4 (2)
C1—S3—C4	91.32 (13)	C9—C10—H10A	109.1
C5—N1—N2	111.8 (2)	C11—C10—H10A	109.1
C5—N1—Ni1	126.77 (17)	C9—C10—H10B	109.1
N2—N1—Ni1	121.46 (14)	C11—C10—H10B	109.1
C6—N2—N1	111.74 (19)	H10A—C10—H10B	107.9
C2—C1—S3	112.9 (2)	C12—C11—C10	114.6 (2)
C2—C1—H1	123.6	C12—C11—H11A	108.6
S3—C1—H1	123.6	C10—C11—H11A	108.6
C1—C2—C3	112.4 (2)	C12—C11—H11B	108.6
C1—C2—H2	123.8	C10—C11—H11B	108.6
C3—C2—H2	123.8	H11A—C11—H11B	107.6
C4—C3—C2	112.9 (2)	C13—C12—C11	113.4 (2)
C4—C3—H3	123.5	C13—C12—H12A	108.9
C2—C3—H3	123.5	C11—C12—H12A	108.9
C3—C4—C5	122.6 (2)	C13—C12—H12B	108.9
C3—C4—S3	110.53 (18)	C11—C12—H12B	108.9
C5—C4—S3	126.78 (19)	H12A—C12—H12B	107.7
N1—C5—C4	130.1 (2)	C14—C13—C12	113.7 (3)
N1—C5—H5	115.0	C14—C13—H13A	108.8
C4—C5—H5	115.0	C12—C13—H13A	108.8
N2—C6—S1	124.49 (19)	C14—C13—H13B	108.8
N2—C6—S2	119.99 (18)	C12—C13—H13B	108.8
S1—C6—S2	115.51 (14)	H13A—C13—H13B	107.7
C8—C7—S2	111.63 (17)	C13—C14—H14A	109.5
C8—C7—H7A	109.3	C13—C14—H14B	109.5
S2—C7—H7A	109.3	H14A—C14—H14B	109.5
C8—C7—H7B	109.3	C13—C14—H14C	109.5
S2—C7—H7B	109.3	H14A—C14—H14C	109.5
H7A—C7—H7B	108.0	H14B—C14—H14C	109.5
C9—C8—C7	109.2 (2)		
			
C5—N1—N2—C6	−179.6 (2)	N1—N2—C6—S1	−1.3 (3)
Ni1—N1—N2—C6	0.4 (3)	N1—N2—C6—S2	177.52 (15)
C4—S3—C1—C2	−1.0 (2)	Ni1—S1—C6—N2	1.4 (2)
S3—C1—C2—C3	0.9 (3)	Ni1—S1—C6—S2	−177.45 (11)
C1—C2—C3—C4	−0.3 (3)	C7—S2—C6—N2	2.3 (2)
C2—C3—C4—C5	176.5 (2)	C7—S2—C6—S1	−178.84 (14)
C2—C3—C4—S3	−0.5 (3)	C6—S2—C7—C8	−177.36 (18)
C1—S3—C4—C3	0.8 (2)	S2—C7—C8—C9	178.55 (18)
C1—S3—C4—C5	−176.0 (2)	C7—C8—C9—C10	−177.8 (2)
N2—N1—C5—C4	−2.1 (4)	C8—C9—C10—C11	176.7 (2)
Ni1—N1—C5—C4	177.9 (2)	C9—C10—C11—C12	−179.8 (2)
C3—C4—C5—N1	178.5 (3)	C10—C11—C12—C13	178.0 (3)
S3—C4—C5—N1	−5.1 (4)	C11—C12—C13—C14	179.1 (3)

Symmetry code: (i) −*x*+1, −*y*+1, −*z*+1.

### Hydrogen-bond geometry (Å, º)

**Table:**

*D*—H···*A*	*D*—H	H···*A*	*D*···*A*	*D*—H···*A*
C2—H2···S1^ii^	0.95	3.00	3.684 (3)	131
C2—H2···S2^iii^	0.95	2.93	3.752 (3)	146
C5—H5···S1^i^	0.95	2.42	3.067 (3)	125
C7—H7*A*···S3	0.99	2.93	3.406 (3)	110

Symmetry codes: (i) −*x*+1, −*y*+1, −*z*+1; (ii) *x*, −*y*+1/2, *z*−1/2; (iii) *x*, −*y*−1/2, *z*−1/2.
